# Detection of novel chromosome-SCC*mec *variants in Methicillin Resistant *Staphylococcus aureus *and their inclusion in PCR based screening

**DOI:** 10.1186/1756-0500-4-150

**Published:** 2011-05-26

**Authors:** Anneke van der Zee, Lieuwe Roorda, Willem DH Hendriks, Jacobus M Ossewaarde, Johannes Buitenwerf

**Affiliations:** 1 Maasstad Laboratory, Molecular Diagnostics Unit, Maasstad Hospital, Olympiaweg 350, 3870HT Rotterdam, The Netherlands; 2 Erasmus Medical Centre, Rotterdam, The Netherlands

## Abstract

**Findings:**

To facilitate automation, a novel DNA extraction method for MRSA was adopted. The MRSA specific chromosome-SCC*mec *PCR was adapted, additional primers were added, and the performance was validated. From various laboratories in The Netherlands we received a total of 86 MRSA clinical isolates, that were negative in commercially available tests. We identified 14 MRSA strains with new variant chromosome-SCC*mec *junctions by sequence analysis. These MRSA strains appeared to carry SCC*mec *sequences with a high degree of homology to SCC regions of *S. epidermidis *and *S. haemolyticus*. All were included for detection in chromosome-SCC*mec *based PCR.

**Background:**

Efficient management of Methicillin Resistant *Staphylococcus aureus *(MRSA) in the hospital is needed to prevent dissemination. It is important that MRSA can be rapidly identified, and effective infection control measures can be initiated. Equally important is a rapid MRSA negative report, especially for patients in isolation. For negative screening we implemented fully automated high through-put molecular screening for MRSA.

**Conclusions:**

Fourteen variant chromosome-SCC*mec *junctions in MRSA, that are not detected in commercially available MRSA detection kits were added to our PCR to detect all currently known variant SCC-*mec *types of MRSA.

## Background

The dissemination of Methicillin Resistant *Staphylococcus aureus *(MRSA) in hospitals is a growing problem worldwide. In The Netherlands, a search and destroy policy is implemented [1]. Patients colonized with MRSA are kept in isolation until they are culture negative. A MRSA negative report can faster be obtained by PCR. Therefore, a molecular approach for negative screening of MRSA was exploited. Molecular detection of the *mecA *gene, which confers resistance to all β-lactams, has often been used in combination with other *S. aureus *specific genes in a multiplex PCR. Genes that are specific for *S. aureus *comprise for example of the sequence published by Martineau et al. [2], the nuclease gene (*nuc*) [3,4], or the coagulase gene (*coa*) [5]. When clinical samples contain a mixture of coagulase negative staphylococci (CNS), methicillin sensitive *S. aureus *(MSSA), and MRSA, a positive *mecA *PCR can be generated by CNS while both MSSA and MRSA generate a positive PCR for the *coa *or the *nuc *gene. Only culturing could confirm MRSA. Another approach for detection of MRSA was presented by a multiplex PCR described by Huletsky et al. [6]. This PCR specifically targets the junction between a conserved open reading frame *orfX *in *S. aureus*, and the staphylococcal cassette chromosome containing the *mecA *gene (SCC*mec*). For MRSA, 8 different types of SCC*mec *elements have been classified [7]. The SCC is known to be a mobile heterogeneous genetic element that integrates site specifically into *orf*X. SCC*mec *is an SCC containing the *mec*A gene. SCC can also be present in CNS, not containing *mecA *but integrated into the analogous chromosomal location. MSSA can contain non-*mecA*-SCC or SCC*mec *elements which have lost the region containing *mecA*. Several commercially available molecular screening tests are based on PCR amplification of the chromosome-SCC*mec *junction.

In this study, a novel DNA extraction method for MRSA was adopted that virtually prevents PCR inhibition. The detection process was fully automated for high through-put of clinical materials. An extra 17 forward primers were added to PCR to detect several newly identified MRSA strains in this study carrying SCC*mec *variants and found to be present in The Netherlands, and possibly elsewhere. An adaptable PCR format is needed for reliable detection of all MRSA.

## Findings

### Implementation and evaluation of *orfX*/SCC*mec *PCR

The PCR as described by Huletsky et al. [6], was slightly adapted (Table 1).

**Table 1:** Primer and probe sequences used in OrfX-SCC PCR

primer/probe	Sequence 5'-3'	Reference
F1	GTCAAAAATCATGAACCTCATTACTTATG	according to Huletsky et al.

F2	AATATTTCATATATGTAATTCCTCCACATCTC	adapted from Huletsky et al.

F3	CTTCAAATATTATCTCGTAATTTACCTTGTTC	adapted from Huletsky et al.

F4	CTCTGCTTTATATTATAAAATTACGGCTG	according to Huletsky et al.

F5	TCACTTTTTATTCTTCAAAGATTTGAGC	adapted from Huletsky et al.

F7	CCATTTCTTCCAAAAAATATATTTACTTTAGTC	This study

F8	TTTCATAATATGTGCTACGCAACCTA	This study

F9	CGAGTTAATTTTTTATTTTAGAGCGCTTAC	This study

F10	CCGCTCCTTTTATATTATACACAACCTATT	This study

F11	GCCATATTAATGCCTCACGAAAC	This study

F12	CATTCATTAACATCGTACTCTGCATTT	This study

F13	TCCCTTTATGAAGCGGCTGAA	This study

F14	AAGCTATAGTTCAGCATTATCGTAAGTTAACT	This study

F15	TGCCAATCACAGTTCAATCAATTATT	This study

F16	TCCTTTCTAATTATATTATGCGCAACCT	This study

F17	ACTCTGATAAGCCATTCATTCATCCA	This study

F18	ACAATCCTAACATAAGATTGTGGCTTT	This study

F20	GCATATTCACTTTGATAAGCCATTCAT	This study

F21	CGGTTCTGATATCTTTTCAACCATT	This study

F23	CCCCTCCCATTAACTCCGTATAT	This study

F24	CCCAAACTCTTAACTTTCTTCAATACATT	This study

F25	TTCTAAGGTAGCTTCCCTTTCAATAATTT	This study

R1	CGTCATTGGCGGATCAAAC	adapted from Huletsky et al.

R2	CGTCATTGGTGGATCAAACG	adapted from Huletsky et al.

probe2	FAM-CACAAGGATGTCTTACAACG-MGB	adapted from Huletsky et al.

probe3	FAM-CACAAGGACGTCTTACAACG-MGB	adapted from Huletsky et al.

probe4	FAM-CACAAAGACGTCTTACAACG-MGB	adapted from Huletsky et al.

To allow PCR detection of more MRSA types, a literature search was conducted. Forward primer F7 was derived from the sequence of *S. aureus *strain JCSC 3624 (WIS), accession number AB121219 [8], and was included in the PCR. Primer F10 was designed based on the sequence of *S. aureus *U10927 [9] (Table 1).

With the expanded *orfX*/SCC*mec *PCR a total of 1906 samples was investigated with high through-put screening; 303 were PCR positive, no inhibition of PCR was found. To verify whether a positive signal was due to viable or dead MRSA, all were cultured; 141 were culture positive, and 22% of 141 were found to be MSSA. The latter may have lost *mecA *regions or contain non-*mecA *SCC elements. All culture positive MRSA were confirmed by *mecA/coa *PCR. Thus, PCR is highly non specific. However, since all suspect samples are subsequently cultured, this is acceptable. With negative screening it is important that the negative predictive value is 100%.

One MRSA isolate found by routine culture was negative in PCR. We analyzed the sequence of this strain (303480, Table 2) by genomic DNA sequencing with primer R1. A sequence of 345 bp was obtained which was aligned to Genbank/EMBL DNA sequences using BLAST. No significant sequence homology was found except that the 35 bp flanking *orfX *showed 97% homology with a repeated sequence found in *S. haemolyticus *(AP006716;bp 52313, and 91034), also present in *S. saprophyticus *(AP08934;bp 50105). A new primer in PCR (primer F8, Table 1) was derived from the obtained sequence. To minimize the chance for more false-negative PCR results for MRSA, other laboratories in The Netherlands were asked for MRSA strains that were negative in commercial molecular tests used by these laboratories. We received a total of 86 clinical isolates or DNA. Whenever a strain was also negative in our *orfX*/SCC*mec *PCR, DNA was sequenced and a new primer was added to PCR. Of 86 strains, another 14 additional forward primers were designed, apart from 2 literature based primers, and 1 primer based on a MRSA strain from our own hospital. All 17 primers were included in PCR to a total of 22 forward primers (Table 1).

**Table 2:** Observed sequence homologies of analyzed MRSA strains from various locations, length of DNA sequence analysis reads, and PCR primers based on these sequences

Strain	Location	bp	Homology (%) with accession number (species) corresponding to bp	primer
02M023064	Breda	222	94% U10927 (*S. aureus*);bp 648-426	F9

JBZ12	Den Bosch	235	93% AB539727 (*S. aureus*);bp 84645-84507	F13

261207168	Enschede	834	91% AB063172 (*S. aureus*); bp 169-879	F15

JBZ33	Den Bosch	396	99% EU272080 (*S. aureus*);bp 794-399	F23

S0121	Utrecht	280	100% EU263618 (*S. aureus*);bp 655-376	F17

CC8	Denmark ( )	422	99% BK001539 (*S. epidermidis*);bp 19437-19016	F20

434-1819	Eindhoven	416	99% BK001539 (*S. epidermidis*);bp 34917-34205	F24

S0089	Utrecht	409	100% AP006716 (*S. haemolyticus*);bp 52718-52358	F16

582	Utrecht	344	98% AP006716 (*S. haemolyticus*);bp 91374-91031	F18

060120	Leiden	912	98% AP006716 (*S. haemolyticus*);bp 30663-29755	F25

303480	Rotterdam	345	CGCAACCTATTTTTTAGTTTTATTTGTGATAtGCT	F8

251110219	Enschede	900	CAACtTATTTTTTAGTTTTATTTGTGATACGCTTCT	F14

JBZ54	Den Bosch	688	No significant homology found	F21

40295861	Breda	417	No significant homology found	F11

40461611	Breda	762	No significant homology found	F12

### Sequence analysis of PCR negative MRSA strains

All 14 strains mentioned above were verified to be MRSA using *mecA/coa *PCR. All were positive in both PCRs. The results of DNA sequence analysis are presented in Table 2. BLAST comparisons were made with the sequences cut off at the *orfX *according to Ito et al. [10]. Within the *orfX *gene, the obtained sequences were highly homologous and consistently aligned to *S. aureus orfX*.

The SCC*mec *of 5 strains showed various degrees of homology (91-100%) with different parts of SCC*mec *sequences of *S. aureus *strains. Two MRSA strains showed 99% homology with *S. epidermidis *SCC, but with different regions. Another 3 strains were homologous to *S. haemolyticus *in their *orfX *flanking sequences. BLAST alignments of strains 303480 and 251110219 showed no significant sequence homologies except for a 35 bp repeat (CAACtTATTTTTTAGTTTTATTTGTGATACGCTTCT)found present in *S. haemolyticus *and *S. saprophyticus*. The *orfX *flanking regions of 3 other strains showed no significant homology to any Genbank/EMBL sequences.

### Comparison of right SSC*mec*-OrfX junctions

**Figure 1:**
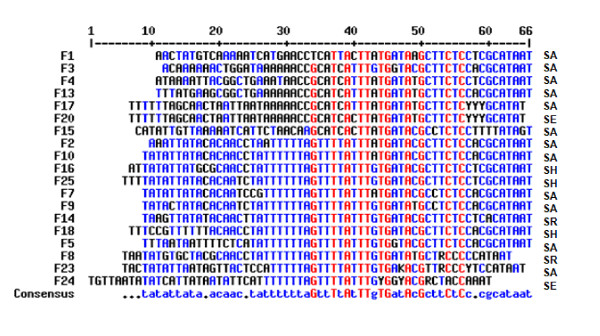
**Alignment of right SCC-OrfX junctions**. Strains are designated by the primers based on their sequences. OrfX starts at bp 55 (according to Ito et al. [10]. SA; *S. aureus*, SE; S*. epidermidis*, SH; *S. haemolyticus*, SR; Staphylococcal repeats. Strains without any homology to known sequences were omitted from the comparison.

The alignment of right SCC*mec*-OrfX junctions is shown in Figure 1. The direct repeat consensus (--A-TT-TGATA-GC-TC, [10]) is largely intact, suggesting that SCC sequences from *S. epidermidis *and *S. haemolyticus *were acquired by recombination rather than by transposition.

## Discussion

The staphylococcal cassette chromosome SCC*mec *is a genetic mobile element that contains the methicillin resistant *mecA *gene [8]. Its site-specific insertion or excision is mediated by cassette chromosome recombinase (ccr) complex and by the presence of direct and inverted repeats at the SCC*mec *extremities. The combination of classes of *mec *gene complex and *ccr *gene complex forms the basis of their classification [10]. At present 8 types of SCC*mec *elements have been classified [7,11]. In addition, SCC*mec *can harbour other drug resistant genes, and insertion sequence elements (*IS*431, *IS*1272), transposons (Tn4001, Tn554) or plasmids (pUB110, pT181). The chromosome flanking regions (junction or J-regions, formerly, junkyard regions) of SCC*mec *are most variable in their DNA sequences.

We adapted the PCR described by Huletsky et al. [6], based on amplification of the chromosome-SCC*mec *junction. We initially included 2 more forward primers to expand detection to MRSA known from literature. After sequence analysis of 14 MRSA strains that were not recognized by the initial primer set, we included additional primers resulting in a megaplex PCR with 14 primers based on sequences of newly identified MRSA with variant SCC*mec *sequences to a total of 21 forward primers. No adverse effects on PCR were seen with this number of primers.

From 3 strains the sequenced SCC*mec *region did not match any known DNA sequence, presuming a non-human relation. In The Netherlands live-stock associated MRSA is increasingly isolated [12]. Reischl et al. [13] have shown that live-stock associated MRSA can be discriminated on the basis of a G→A mutation in *orfX*. Based on the finding that these strains were only positive in PCR when the probe harbouring this mutation (probe 2) was used, we confirmed that these 3 strains were most probably live-stock associated MRSA.

In the SCC*mec *of the other new strains, repeated sequences as *IS*431 and *IS*1272 were found. IS elements are mobile and can move by transposition. The similar but not identical repeats found in strain 303480 and 251110219, might be related to an *IS*1272 encoded truncated transposase as was indicated by BLAST homology. This suggested that the variant SCC*mec *sequences resulted from transposition. Therefore the right chromosome-SCC*mec *junctions carried by the new MRSA strains were compared. The consensus represented the direct repeat (DR) involved in insertion and excision of SCC*mec*. Consequently, the variant SCC*mec *sequences were acquired by recombination rather than transposition. Mongkolrattanothai et al. [14], reported a non-*mecA*-SCC element present in *S. epidermidis *with 3 DR highly homologous to *S. aureus*, 2 at the extremities of the element and 1 in the middle. The 2 MRSA strains carrying different SCC homologs of *S. epidermidis *in this study, were each highly homologous to the SCC described by Mongkolrattanothai [14], but represented the sequence on either side of the middle DR. This finding may corroborate the recombination theory, regardless of the orientation of the inserted SCC in the 2 MRSA strains. Whether *mecA *is carried on the acquired SCC from *S. epidermidis *of the MRSA strains found in our study, as well as SCC typing, remains a topic for future studies.

In conclusion, it appears that MSSA can convert to MRSA by acceptance of SCC sequences from CNS. This process may be driven by selection pressure of antibiotics. Indeed, an isogenic pair of MSSA and MRSA was recently described that supports the occurrence of horizontal transfer of SCC*mec *between Staphylococcal species [15]. The 86 strains not detected as being MRSA in other laboratories by commercially available tests raises concern about their performance [16,17]. Exploiting the SCC*mec *junction as target in PCR used for detection of MRSA requires continuous awareness of possible variants. PCR based on *mecA*/*nuc *gene does not seem to present an acceptable alternative because the *nuc *gene may be absent as has been found earlier [18]. The *mecA*/*coa *PCR, combined with chromosome-SCC*mec *junction PCR identifies other MRSA variants that may arise.

## Conclusions

We identified 14 new variant chromosome-SCC*mec *junctions in MRSA, that are not detected in commercially available MRSA detection kits. We adapted our PCR to detect all known variant chromosome-SCC-*mec *types of MRSA.

Fully automated high through-put detection and robust DNA extraction support an adaptable PCR format for detection of these newly identified MRSA strains. Moreover, this system can easily be expanded with novel PCR primers when new variants of chromosome-SCC*mec *types in MRSA may arise.

## Methods

### Clinical samples

Samples were taken from nose, throat, and perineum, and if appropriate also from wounds, sputum, and catheters (Transwab, Medical Wire & Equipment Co. Ltd., Corsham, Wilts., England) in the context of patient and personnel screening as required by the Dutch policy for MRSA control. Since no extra action or sampling was requested than the medically indicated, informed consent was not asked and no ethical approval was required, in conformity to the guidelines of the Dutch Central Committee on Research involving Human Subjects. Swabs were inoculated into 5 ml phenyl mannitol broth (PHMB) containing ceftizoxime and aztreonam [19] for overnight incubation. After at least 18 hours of incubation, PHMB broth was subjected to PCR.

### Automated DNA isolation, PCR detection, and data output by MRSA-screen with PCR amplification of OrfX-SCC*mec *junction

PHMB tubes were placed into the MultiPROBE II PLUS HT Expanded pipetting robot (PerkinElmer Life and Analytical Sciences). This system is equipped with a bar-code reader and automated plate sealer (RoboSeal). This system handles the DNA extraction, PCR assay set-up, and sealing of the plate. Next, the plates are automatically transferred to a real-time ABI 7900HT thermocycler (Applied Biosystems, Nieuwerkerk a/d IJssel, The Netherlands). Thus, barcode scanning, DNA extraction, pipetting, sealing of the 384-well PCR Plate, creating a run file and starting the PCR, were done by the system without interference or need of presence of a technician. Results were automatically transferred to the Laboratory Information System.

Software was programmed by PerkinElmer to perform the following steps. After barcode reading, 5 ml of saline was added and mixed by pipetting. This 1:1 dilution served to elevate the fluid level and to prevent the 9.5 cm long probes touching the swabs in the 15 cm long tubes.

Template DNA was prepared by using the Extract-N-Amp™ Plant PCR Kit (SIGMA, Munich, Germany). In short, 50 μl sample was mixed with 100 μl SIGMA Extraction Solution (E7526). The mixture was incubated at 95 °C for 10 minutes, cooled to room temperature, 100 μl SIGMA Dilution Buffer (D5688) was added and mixed. This DNA sample is PCR ready when used in combination with the SIGMA Extract-N-Amp PCR ReadyMix™ (E3004).

Primers and probes for detection of the OrfX-SCC*mec *junction were used in one primer-probe mixture with the following concentrations; 600 nM for each F and R primer, and 120 nM for each probe (Table 1). Each sample was tested with this primer-probe mixture. Amplification reactions were performed in a volume of 20 μl, with 10 μl SIGMA Extract-N-Amp PCR Reaction Mix™ (including 4% Reference Dye) in 6 μl primer-probe mix and 4 μl of the DNA sample. Amplification consisted of 3 min at 95 °C followed by 44 cycles of 15 s at 95 °C and 60 s at 60 °C. In each run a MRSA positive and a negative control was included. A sample was regarded suspect for MRSA in the MRSA-screen if the PCR was positive. Positive samples were subsequently cultured on blood agar plates.

### *MecA/coa *gene PCR

The *mecA *PCR was carried out as described [4], with 800nM of each primer. PCR for *coa *was as described previously [5]. The concentration of primers Coag2: CGAGACCAAGATTCAACAAG, and Coag3: AAAGAAAACCACTCACATCA was 800nM each. *MecA/coa *PCR was used to confirm MRSA isolates. Results of *mecA *and *coa *PCR were analyzed using agarose gel electrophoresis.

### DNA sequence analysis

Sequence analysis was performed by Baseclear, Leiden, The Netherlands on total chromosomal DNA with primer R1 (Table 1). Partial sequences of variant SCC*mec *have not yet been submitted but will be classified in further studies according to the recommendations of the International working group on the classification of staphylococcal cassette chromosome elements [11].

## Competing interests

The authors declare that they have no competing interests.

## Authors' contributions

AZ drafted the manuscript and carried out the analysis of data. LR carried out the molecular genetic studies, and participated in the design of PCR. WDHH and JB conceived of the study and participated in its design. JMO helped to draft the manuscript. All authors read and approved the final manuscript.

## References

[B1] Vos MC Ott A Verbrugh HA Successful Search-and-Destroy Policy for Methicillin-Resistant Staphylococcus aureus in The Netherlands J Clin Microbiol 2005 43 4 2034 2035 10.1128/JCM.43.4.2034-2035.2005 15815056 PMC1081402

[B2] Martineau F Picard FJ Roy PH Ouellette M Bergeron MG Species-specific and ubiquitous-DNA-based assays for rapid identification of *Staphylococcus aureus* J Clin Microbiol 1998 36 618 623 9508283 10.1128/jcm.36.3.618-623.1998 PMC104596

[B3] Francois P Bento M Renzi G Harbarth S Pittet D Schrenzel J Evaluation of Three Molecular Assays for Rapid Identification of Methicillin-Resistant Staphylococcus aureus J Clin Microbiol 2007 45 2011 2013 10.1128/JCM.00232-07 17428926 PMC1933053

[B4] Geha D J Uhl J R Gustaferro C A Persing D H Multiplex PCR for identification of methicillin-resistant staphylococci in the clinical laboratory J Clin Microbiol 1994 32 1768 1772 7929772 10.1128/jcm.32.7.1768-1772.1994 PMC263789

[B5] Goh S H Byrne SK Chow AW Molecular typing of *Staphylococcus aureus *on the basis of coagulase gene polymorphisms J Clin Microbiol 1992 30 1642 1645 1352784 10.1128/jcm.30.7.1642-1645.1992 PMC265357

[B6] Huletsky A Giroux R Rossbach V Gagnon M Vaillancourt M Bernier M Gagnon F Truchon K Bastien M Picard FJ van Belkum A Ouellette M Roy PH Bergeron MG New real-time PCR assay for rapid detection of methicillin resistant *Staphylococcus aureus *directly from specimens containing a mixture of staphylococci J Clin Microbiol 2004 42 1875 1884 10.1128/JCM.42.5.1875-1884.2004 15131143 PMC404602

[B7] Chongtrakool P Ito T Ma X X Kondo Y Trakulsomboon S Tiensasitorn C Jamklang M Chavalit T Song JH Hiramatsu K Staphylococcal Cassette Chromosome *mec *(SCC*mec*) Typing of Methicillin-Resistant *Staphylococcus aureus *Strains Isolated in 11 Asian Countries: a Proposal for a New Nomenclature for SCC*mec *Elements Antimicrob Agents Therap 2006 50 3 1001 1012 10.1128/AAC.50.3.1001-1012.2006 PMC1426434 16495263

[B8] Ito T Katayama Y Asada K Mori N Tsutsumimoto K Tiensasitorn C Hiramatsu K Structural comparison of three types of staphylococcal cassette chromosome *mec *integrated in the chromosome in methicillin-resistant *Staphylococcus aureus* Antimicrob Agents Therap 2001 45 1323 1336 10.1128/AAC.45.5.1323-1336.2001 PMC90469 11302791

[B9] Lin WS Cunneen T Lee CY Sequence analysis and molecular characterization of genes required for the biosynthesis of type 1 capsular polysaccharide in Staphylococcus aureus J Bacteriol 1994 176 22 7005 7016 7961465 10.1128/jb.176.22.7005-7016.1994 PMC197074

[B10] Ito T Ma XX Takeuchi F Okuma K Yuzawa H Hiramatsu K Novel type V staphylococcal cassette chromosome *mec *driven by a novel cassette chromosome recombinase, *ccrC* Antimicrob Agents Therap 2004 48 2637 2651 10.1128/AAC.48.7.2637-2651.2004 PMC434217 15215121

[B11] International working group on the classification of staphylococcal cassette chromosome elements (IWG-SCC) Classification of Staphyloccoccal Casette chromosome mec (SCCmec): Guidelines for reporting novel SCCmec elements Antimicrob Agents Therap 2009 53 12 4961 4967 10.1128/AAC.00579-09 PMC2786320 19721075

[B12] De Neeling AJ van den Broek MJ Spalburg EC van Santen-Verheuvel MG Dam-Deisz WDC Boshuizen HC van de Giessen AW van Duijkeren E Huijsdens XW High prevalence of methicillin resistant Staphylococcus aureus in pigs Vet Microbiol 2007 122 3-4 366 72 10.1016/j.vetmic.2007.01.027 17367960

[B13] Reischl U Frick J Hoermansdorfer S Melzl H Bollwein M Linde HJ Becker K Köck R Tuschak C Busch U Sing A Single-nucleotide polymorphism in the SCCmec-orfX junction distinguishes between livestock-associated MRSA CC398 and human epidemic MRSA strains Euro Surveill 2009 14 49 19436 20003904

[B14] Mongkolrattanothai K Boyle S Murphy TV Daum RS Novel non-*mecA*-containing staphylococcal chromosomal cassette composite island containing *pbp4 *and *tagF *genes in a commensal staphylococcal species: A possible reservoir for antibiotic resistance islands in *Staphylococcus aureus* Antimicrob Agents Therap 2004 48 1823 1836 10.1128/AAC.48.5.1823-1836.2004 PMC400556 15105141

[B15] Bloemendaal ALA Brouwer EC Fluit AC Methicillin Resistance transfer from Staphylococcus epidermidis to Methicillin-susceptible Staphylococcus aureus in a patient during antibiotic therapy PLoS ONE 2010 5 7 e11841 10.1371/journal.pone.0011841 20686601 PMC2912275

[B16] Bartels MD Boye K Rohde SM Larsen AR Torfs H Bouchy P Skov R Westh H A common variant of staphylococcal cassette chromosome mec type IVa in isolates from Copenhagen, Denmark, is not detected by the BD GeneOhm methicillin-resistant Staphylococcus aureus assay J Clin Microbiol 2009 47 5 1524 7 10.1128/JCM.02153-08 19297600 PMC2681823

[B17] Ornskov D Kolmos B Bendix Horn P Nederby Nielsen J Brandslund I Schouenborg P Screening for methicillin-resistant Staphylococcus aureus in clinical swabs using a high-throughput real-time PCR-based method Clin Microbiol Infect 2008 14 22 28 18034860 10.1111/j.1469-0691.2007.01880.x

[B18] van Leeuwen W Roorda L Hendriks W Francois P Schrenzel J A nuc-deficient meticillin-resistant Staphylococcus aureus strain FEMS Immunol Med Microbiol 2008 54 157 10.1111/j.1574-695X.2008.00478.x 18811719

[B19] Wertheim H Verbrugh HA van Pelt C de Man P van Belkum A Vos MC Improved detection of methicillin-resistant *Staphylococcus aureus *using phenyl mannitol broth containing aztreonam and ceftizoxime J Clin Microbiol 2001 39 2660 2662 10.1128/JCM.39.7.2660-2662.2001 11427589 PMC88205

